# Fermentation of Ammonia Fiber Expansion Treated and Untreated Barley Straw in a Rumen Simulation Technique Using Rumen Inoculum from Cattle with Slow versus Fast Rate of Fiber Disappearance

**DOI:** 10.3389/fmicb.2016.01839

**Published:** 2016-11-16

**Authors:** Candace L. Griffith, Gabriel O. Ribeiro, Masahito Oba, Tim A. McAllister, Karen A. Beauchemin

**Affiliations:** ^1^Lethbridge Research and Development Centre, Agriculture and Agri-Food Canada, LethbridgeAB, Canada; ^2^Agricultural, Food and Nutritional Science, University of Alberta, EdmontonAB, Canada; ^3^CAPES Foundation, Ministry of Education of BrazilBrasília, Brazil

**Keywords:** fiber digestion, pre-treatment, real-time PCR, straw, microbiome, ammoniation

## Abstract

The purpose of this study was to determine the effect of rumen inoculum from heifers with fast vs. slow rate of *in situ* fiber digestion on the fermentation of complex versus easily digested fiber sources in the forms of untreated and Ammonia Fiber Expansion (AFEX) treated barley straw, respectively, using an artificial rumen simulation technique (Rusitec). *In situ* fiber digestion was measured in a previous study by incubating untreated barley straw in the rumen of 16 heifers fed a diet consisting of 700 g/kg barley straw and 300 g/kg concentrate. The two heifers with fastest rate of digestion (Fast ≥ 4.18% h^-1^) and the two heifers with the slowest rate of digestion (Slow ≤ 3.17% h^-1^) were chosen as inoculum donors for this study. Two Rusitec apparatuses each equipped with eight fermenters were used in a completely randomized block design with two blocks (apparatus) and four treatments in a 2 × 2 factorial arrangement of treatments (Fast or Slow rumen inoculum and untreated or AFEX treated straw). Fast rumen inoculum and AFEX straw both increased (*P* < 0.05) disappearance of dry matter (DMD), organic matter, true DMD, neutral detergent fiber, acid detergent fiber, and nitrogen (N) with an interactive effect between the two (*P* < 0.05). Fast rumen inoculum increased (*P* > 0.05) methane production per gram of digested material for both untreated and AFEX straw, and reduced (interaction, *P* < 0.05) acetate: propionate ratio for untreated straw. Greater relative populations of *Ruminococcus albus* (*P* < 0.05) and increased microbial N production (*P* = 0.045) were observed in Fast rumen inoculum. AFEX straw in Fast inoculum had greater total bacterial populations than Slow, but for untreated straw this result was reversed (interaction, *P* = 0.013). These findings indicate that differences in microbial populations in rumen fluid contribute to differences in the capacity of rumen inoculum to digest fiber.

## Introduction

Variation among beef cattle in residual feed intake (Koch et al., 1963; Herd et al., 2004), feed efficiency, feeding behavior, metabolic rate, and methane production (Nkrumah et al., 2006) has been well documented and thus it is logical to infer that variability in rumen fermentation occurs as well. It has recently been established that around the world, the rumen of cattle has the same core microbiome at the genus level (Jami and Mizrahi, 2012; Henderson et al., 2015), with abundance and types of microbial species varying among individual animals. Although there is variation among microbial species, there seem to be overall functional similarities of rumen microbial communities (Galbraith et al., 2004; Jami and Mizrahi, 2012). Weimer et al. (2010) reported that when >95% rumen contents were transferred between two cattle fed the same diet, with differing host-specific microbial populations, the populations reverted back to those possessed by the original host within 14 and 61 days. This suggests the existence of a hologenome, where interactions between host and microbial genetic components result in the establishment of a unique microbiota that helps regulate host physiological responses (Rosenberg et al., 2010). For example, Jami et al. (2014) reported that increased milk fat production in dairy cows was strongly correlated to an increase in the ratio of *Firmicutes* to *Bacteroidetes* in rumen contents. A decrease in *Bacteroidetes* relative to *Firmicutes* has been found in obese mice and is connected to an increase in blood and tissue fat (Turnbaugh et al., 2006). In line with that, greater feed efficiency has been reported in cattle with a less diverse rumen microbiome due to less complex metabolic pathways (Shabat et al., 2016). Exploration of the differences between cattle due to their inherent gut microbiomes and the potential differences in digestive capacity is of interest. There is a paucity of information that links individual variation in digestion efficiency and the rumen microbiome. Optimizing the ruminal microbiome of individual animals to improve digestive function could improve fiber digestion in the rumen and decrease cost of animal production.

Another potential avenue for mitigating feed costs is the use of less costly agricultural residues as ruminant feed sources. Straw is one such abundant byproduct, but its total digestible nutrient (TDN) content is low [40–46% of dry matter (DM); Kopp, 2003], limiting its use in ruminant diets. To this end, much research has examined the possible use of alkali pre-treatments such as ammoniation as a means of enhancing the digestibility of neutral detergent fiber (NDF) in the rumen (Hendriks and Zeeman, 2009; Alvira et al., 2010; Talebnia et al., 2010; Abdel-Aziz et al., 2015). Ammoniation of straw has been shown to disrupt hemicellulose-lignin bonds and cellulose crystallinity to allow enzymes access and increase hydrolysis of hemicellulose and cellulose. However, traditional ammoniation methods pose potential health hazards and a large portion of the ammonia is volatilized (Freney et al., 1983; Rasby et al., 1989). Efficiency of ammoniation treatment has been improved with the advent of ammonia freeze explosion (Dale and Moreira, 1982), later termed Ammonia Fiber Expansion (AFEX^TM^). AFEX uses moisture and high pressure during ammonia treatment, with a subsequent pressure release and ammonia removal (Campbell et al., 2013). Bals et al. (2010) found that AFEX was far more effective as it increased digestion of late harvest switchgrass by 206% as compared to a 56% increase with traditional ammoniation methods. Using AFEX and untreated barley straw in the study allowed us to examine the effect of inoculum source on digestion of easily accessible fiber source and a more complex fiber source, while using the same feed source.

The *in situ* method (Ørskov and McDonald, 1979) is widely used to characterize fiber digestion in the rumen. As this method involves measuring fiber digestion at different time points, it is possible to estimate the rate of fiber digestion in the rumen. Rates of fiber degradation vary among animals and may be influenced by a number of host factors such as rate of passage, rumen capacity, and saliva production. Therefore in order to examine whether these differences in rate of digestion are related to differences in microbial populations, the rumen simulation technique (Rusitec; Czerkawski and Breckenridge, 1977) is well suited. The Rusitec affords strict control of saliva infusion, amount of feed, time of feeding, temperature, while allowing for measurement of rumen fermentation end products, such as methane (CH_4_), volatile fatty acids (VFA), microbial populations, and pH. Controlling for physiological components such as saliva production and rate of passage allows for a focused investigation of differences in microbial populations (i.e., inoculum sources) while allowing multiple runs simultaneously, simulating multiple cows with the same inoculum.

The objective of this study was to use the Rusitec system to determine whether AFEX treatment improves the ruminal digestibility of barley straw, and whether the extent of this improvement varies among heifers with fast or slow rate of degradation of untreated straw NDF. It was hypothesized that AFEX treatment would increase digestibility of barley straw and that inoculum from heifers with fast rate of degradation would degrade both straws more completely in a 48 h time period than those with a slow rate of degradation.

## Materials and Methods

The experiment was conducted at Agriculture and Agri-Food Canada in Lethbridge, AB, Canada. The experiment was approved by the Lethbridge Research and Development Centre Animal Care Committee and cattle were cared for following the guidelines of the (Canadian Council on Animal Care [CCAC], 2009).

### Experimental Design and Treatments

Two Rusitec apparatuses, each equipped with eight fermenters, were used (*n* = 4 fermenters per treatment) and the experiment was conducted over a period of 15 days with 8 days of adaptation and 7 days of sample collection. The experiment was a completely randomized block design with a 2 × 2 factorial arrangement of treatments; two sources of inoculum (slow or fast rate of NDF disappearance) and two substrates (untreated or AFEX treated barley straw diet). Inoculum from heifers with slow and fast rate of NDF disappearance was obtained by pooling rumen inoculum from two heifers each chosen based on their rate of NDF disappearance (*k*_d_) of barley straw measured *in situ*.

Inoculum donors were preselected by incubating untreated, ground (2-mm) barley straw in duplicate in the rumen of 16 cannulated Angus × Hereford beef heifers fed 700 g/kg untreated barley straw and 300 g/kg concentrate (DM basis) consisting of 666 g/kg dried distillers grains (DDGS), 267 g/kg canola meal, 57 g/kg supplement, and 10 g/kg urea. Barley straw was incubated in the rumen of each heifer for 0, 4, 8, 12, 24, 48, 96, and 120 h during a single incubation period. Bags used for incubation were 10 × 20 cm Ankom bags (R1020, ANKOM Technology, Macedon, NY, USA, 50 micron porosity) with 6.0 g (±0.05g) of feed per bag. Ten minutes prior to insertion into the rumen bags were submerged in 39°C water. Bags were inserted into the rumen 1 h after feeding, and removed after the appropriate amount of time. Duplicate Ankom bags were placed inside larger mesh bags (30 × 30 cm) which were placed into the rumen through the cannula and fully submerged. No microbial contamination correction was performed, as this contamination was assumed to be similar between heifers. Disappearance of NDF was calculated for each time point for each heifer and the rate of NDF disappearance in percent per hour (*k*_d_) was estimated by fitting the data to the following model (McDonald, 1981):

$$\begin{array}{c} P=a+b(1-e^{-k_{d}(t-L)}), \end{array}$$

where *P* is extent of degradation at time *t, a* is the soluble or washout fraction, *b* is the potentially digestible fraction, and *L* is the lag time. Lag time measurements are subject to error, and retention time varies by animals, therefore, *k*_d_ was chosen as the variable for animal selection. Heifers were then ranked from slow to fast based on *k*_d_ and the two animals with the fastest and the two with the slowest rates of disappearance were chosen for this study (Fast ≥ 4.18% h^-1^ vs. Slow ≤ 3.17% h^-1^; **Table 1**).

**Table 1:** *In situ* kinetics of NDF disappearance (NDFD) for Fast^1^ and Slow heifers.

Animal	*k*_d_^2^ (%/h)	Lag (h)	a (%)^3^	b (%)	24 h NDFD^4^	48 h NDFD
Fast 1	4.32	2.80	0.90	58.5	38.6	49.1
Fast 2	4.18	1.67	0.90	56.7	35.7	48.5
Slow 1	3.17	4.13	2.60	56.1	27.4	46.7
Slow 2	2.88	1.11	0.90	61.1	24.4	48.4

*^1^Fast refers to inoculum from animals with fast rate of NDFD; slow refers to animals with slow rate of NDFD.*

*^2^*k*_*d*_ = rate of disappearance per hour based on disappearance of barley straw measured *in sacco.**

*^3^‘a’ fraction is the percentage of washout from the initial substrate; ‘b’ is the percentage potentially degraded in the rumen over 120 h.*

*^4^24 h and 48 h observed NDFD.*

Ammonia Fiber Expansion treatment was performed by Michigan Biotechnology Institute (Lansing, Michigan, USA) using a pair of packed bed AFEX reactors as described by Campbell et al. (2013). Briefly, barley straw was ground through a 30.5 mm screen and packed into stainless steel baskets at a density of 100 kg/m^2^. Baskets were then inserted into a reactor tube where they were pre-steamed in order to displace air and raise the temperature to between 80–85°C. Vapor ammonia was applied at a rate of 80–100 g/min to a level of 1 kg ammonia per kilogram dry straw and a maximum pressure of 200 psi and left for 30 min to soak. Pressure was released, and residual ammonia was stripped by steam stripping and vaporized at atmospheric pressure before repressurized and charged to the next reactor by an ammonia compressor.

### Substrate Processing

Substrates (untreated and AFEX barley straw; **Table 2**) were ground through a 4-mm screen using a Wiley mill (standard model 4; Arthur H. Thomas Co., Philadelphia, PA, USA) and particle size distribution was assessed by sieving 50 g of feed for 5 min on a Ro Tap particle separator (model RX-29; W.S. Tyler, Mentor, OH, USA) equipped with four screens (1,180, 850, 600, and 300 μm) and a bottom pan. Because AFEX straw had a greater percentage of smaller particles because it shattered more than untreated straw, the untreated straw was further ground through a 2-mm screen. To ensure that both substrates had the same particle size distribution each substrate was reconstituted from the sieved fractions to have the following particle size distribution: 100 g/kg > 1,180 μm; 200 g/kg < 1,180 μm and > 850 μm; 350 g/kg < 850 μm and > 600 μm; and 350 g/kg < 600 μm and > 300 μm. The fines (<300 μm) were removed from both substrates to prevent wash out from the bags in fermenters. The same concentrate that was fed to the heifers was ground through a 2-mm screen. Samples were mixed thoroughly and weighed separately into bags with a pore size of 50 μm. Bags used for concentrate were 5 × 10 cm (R510, ANKOM Technology, Macedon, NY, USA); bags used for straw were 10 × 20 cm (R1020, ANKOM Technology, Macedon, NY, USA).

**Table 2:** Ingredient and chemical composition of substrates.

	Ingredients		
Item (g/kg DM)	AFEX barley straw^1^	Untreated barley straw	Concentrate^2^
DM	935	924	905
OM	940	928	905
N	16	7	59
CP	99	43	366
NDF	666	804	357
ADF	488	456	145

*^1^Values for sieved and reconstructed AFEX and untreated straw.*

*^2^Comprised of 66.7% dried distillers grains solids, 26.6% canola meal, 5.7% supplement, 1% urea.*

### Rumen Simulation Technique

Inoculum was collected 1 month after *k*_d_ was measured. Animals were maintained on the same diet of 700 g/kg barley straw and 300 g/kg pelleted concentrate (DM basis) in the interim. Inoculum was obtained from the four selected ruminally cannulated beef heifers 2 h after feeding. Rumen fluid and solid contents were pooled for the two heifers with fast and for the two with slow rates of NDF disappearance. Rumen fluid was filtered through four layers of cheesecloth into insulated thermoses and transported to the laboratory.

Treatments were randomly assigned to 900-mL fermenters so that both Rusitec systems had two replicates per treatment with four replicates per treatment overall. Each fermenter had a buffer input and eﬄuent output port. Fermenters were maintained at 39°C by immersion in a water bath. Each fermenter was filled with 180 mL pre-warmed artificial saliva (pH = 8.2; McDougall, 1948) modified to contain 0.3 g/L of (NH_4_)_2_SO_4_, and 720 mL of strained rumen fluid. Three labeled bags were placed in each fermenter, one containing 10 g of solid rumen digesta, one containing 7 g of barley straw (AFEX or untreated), and one containing 3 g of concentrate. The relative amounts of straw and concentrate were similar to that in the diets fed to the donor heifers. After 24 h, the bag containing rumen digesta was removed and replaced by two bags, one containing 7 g barley straw, and the other containing 3 g concentrate. Thereafter one bag containing concentrate and one bag containing straw were replaced at the same time daily so that each bag remained in the fermenter for 48 h. Bag were exchanged under a stream of O_2_-free CO_2_. The artificial saliva was continuously infused into the fermenters at a rate of 2.9%/h (replacing 70% of the fermenter volume each day). Eﬄuent was collected in a 1 L flask, and gas was collected in a 2 L bag (Curity^®^; Conviden Ltd., Mansfield, MA, USA) attached to the eﬄuent flask. Every day at the time of feed bag exchange, rumen fluid pH, total gas production, and eﬄuent volume were measured.

### Dry Matter and Organic Matter Disappearance

Dry matter disappearance (DMD) and organic matter (OM) disappearance (OMD) at 48 h were determined on days 9–11 and 13–15. Feed bags were removed from each fermenter, washed in cold running water until the water was clear, and dried at 55°C for 48 h. To ensure sufficient sample for chemical analysis, concentrate samples were pooled in groups of 3 days by fermenter for days 9–11 and 12–15. Forage and pooled concentrate samples were ground through a 1-mm screen prior to chemical analysis.

### Fermentation Metabolites

Just prior to feed bag exchange, total gas production from each fermenter was measured daily on days 9–15 using a gas meter (Model DM3A, Alexander–Wright, London, UK). Gas samples (20-mL) were collected from the septum of the collection bags using a 26-gauge needle and transferred to 6.8-mL evacuated exetainers (Labco Ltd., Wycombe, Buckinghamshire, UK). Samples were stored at room temperature until the end of the experiment when they were analyzed for CH_4_.

At the time of feed bag exchange, 2.5-mL subsamples of liquid were collected for VFA and NH_3_N analysis from fermenters on days 11–14. Samples were placed in 5-mL scintillation vials containing 0.5 mL of 25% (w/w) metaphosphoric acid and immediately frozen at -20°C until VFA analysis. For NH_3_N analysis, subsamples were placed in scintillation vials containing 0.5 mL of 1% sulfuric acid for NH_3_N, and then frozen at -20°C until analysis. The concentrations of VFA and NH_3_N (mmol/L) were multiplied by the outflow rate of fluid infused to the vessels (L/day) to determine VFA and NH_3_N production (mmol/d).

### Microbial Protein Synthesis

From day 7 until the end of the experiment, the McDougall’s buffer was modified by replacing (NH_4_)_2_SO_4_ with 0.3 g/L ^15^N-enriched (NH_4_)_2_SO_4_ (Sigma Chemical Co., St Louis, MO, USA; minimum ^15^N enrichment 10.01 atom%; Pilgrim et al., 1970). On days 13–15, the 24 h accumulation of eﬄuent in each flask was preserved with 20% (wt/vol) sodium azide (3 mL) and 40 mL of eﬄuent was subsampled for isolation of bacteria associated with the liquid fraction. The 48 h bag residues were processed to obtain feed particle associated (FPA) and feed particle bound (FPB) bacterial fractions. Bags were removed from the fermenter, gently squeezed and then placed into a plastic bag with 20 mL of McDougall’s buffer and processed for 60 s at 230 rpm in a Stomacher 400 laboratory paddle blender (Seward Medical Ltd., London, UK). Processed liquid was gently squeezed out, decanted and retained in a 50 mL falcon tube. Bags were washed twice more with 10 mL of buffer in each wash and each time the buffer was retained to estimate FPA bacterial fraction. Washed solid feed residues were considered to represent the FPB bacterial fraction.

The eﬄuent liquid samples were then processed by centrifuging at 20,000 × *g* for 30 min at 4°C and the resulting pellet was centrifuged three times at 20,000 × *g* for 30 min at 4°C after washing with McDougall’s buffer. Pellet was re-suspended in distilled water, frozen at -20°C, lyophilized and ball-ground for N and ^15^N analysis. The FPA bacterial samples collected after stomaching were centrifuged (500 × *g*, 10 min, 4°C), with the resulting supernatant subsequently centrifuged (20 000 × *g*, 30 min, 4°C). The resulting pellet was washed three times as described for eﬄuent pellets. The pellet was re-suspended in distilled water, frozen at -20°C, lyophilized and ball-ground for N and ^15^N analysis and 16S rRNA quantification. The washed, solid feed residues, containing the FPB bacterial fraction were dried at 55°C for 48 h, weighed for DM determination and then ground and analyzed for N and ^15^N concentrations.

### Protozoa

Protozoa counts were determined in the fermenters on days 9, 12, and 15. Rumen fluid from each fermenter was collected by gently squeezing the 48 h forage and concentrate bags. The fluid from the forage and concentrate bags were pooled by fermenter and 5.0 mL of the rumen fluid was preserved in 5.0 mL of methyl green formalin-saline solution (Ogimoto and Imai, 1981). Protozoa samples were stored in the dark at room temperature until counted. Protozoa were enumerated by light microscopy using a Levy-Hausser hemacytometer (Hausser Scientific, Horsham, PA, USA). Each sample was counted twice and if the duplicates differed by more than 10%, counts were repeated. Protozoa genera were not characterized as protozoa numbers were very low in the Rusitec making it difficult to accurately evaluate protozoa populations.

### DNA Extraction and 16S rRNA Copy Quantification

DNA was extracted from all ground FPA samples using a Qiagen QIAmp Stool Mini kit (Qiagen Inc., Valencia, CA, USA), slightly modified to improve DNA extraction from Gram-positive bacteria. Briefly, 30 mg of sample was added to 1.4 mL Buffer ASL, stool lysis buffer, and vortexed until thoroughly homogenized (∼1 min). The solution was then pipetted into a new tube containing sterile zirconia beads (0.3 g, 0.1 mm; 0.1 g, 0.5 mm) and homogenized for 3 min at 30/s on a Qiagen Tissue Lyser II (Yu and Morrison, 2004). Samples were then mixed at 700 rpm while heated at 95°C for 5 min. Samples were vortexed briefly and centrifuged at 13,200 rpm for 1 min. The supernatant was separated, added to an inhibitEX tablet and the Qiagen Stool Mini Kit protocol was followed. Total DNA was quantified using PicoGreen with a NanoDrop 3300 fluorometer, normalized to 20 ng/μL, and run on a gel to check for quality.

Using previously described primers and annealing temperatures, qPCR was performed to determine the relative abundance of the following fibrolytic bacteria: *Ruminococcus albus* (Wang et al., 1997)*, Fibrobacter succinogenes, Ruminococcus flavefaciens, Selenomonas ruminantium, Prevotella bryantii* (Tajima et al., 2001), and total bacterial 16S rRNA (Oss et al., 2016).

### Sample analysis

Samples of feed and feed fermentation residues were analyzed for analytical DM by drying 1.0 g (± 0.05 g) of each sample for 2 h at 135° C using a forced air oven. Samples were ashed at 550° C for 5 h to estimate OM. NDF inclusive of ash (NDF) and acid detergent fiber (ADF) were analyzed by the sequential method with the ANKOM200 Fiber Analyzer using reagents as described by Van Soest et al. (1991). Sodium sulphite and α-amylase were used during NDF determination. Total N concentration and atom per cent excess (APE) of ^15^N was determined using a mass spectrometer (Ribeiro et al., 2015). Concentration of CH_4_ in the gas samples was determined using a Varian gas chromatograph equipped with a GS-Carbon-PLOT 30 m × 0.32 mm × 3 μm column and thermal conductivity detector (Agilent Technologies Canada, Inc. Mississauga, ON, Canada). The oven temperature was set at 35°C with an injector temperature of 185°C (1:30 split, 250 μL injector volume) a detector temperature of 150°C and helium (27 cm/s) as the carrier gas. Ammonia was analyzed using the modified Berthelot method as described by Rhine et al. (1998) and VFA were analyzed by gas chromatography as described by Wang et al. (2001).

### Calculations and Statistical Analysis

True dry matter disappearance was determined as DMD adjusted for microbial DM: initial sample weight – (final sample weight-microbial DM)/initial sample weight.

Total eﬄuent microbial N (MN) production (mg/day) was calculated using the N concentration (%) determined for the microbial pellet, multiplied by the microbial weight in the total eﬄuent (mg/day). Microbial weight in the total eﬄuent was calculated by multiplying daily eﬄuent production (mL) by the microbial density (mg/mL) in the 40 mL subsample. Microbial N production from FPA fraction was calculated by multiplying the N concentration (%) in the FPA microbial pellet by the microbial weight of the FPA fraction (mg/day). FPB MN production (mg/day) from straw and concentrate fractions were calculated using the following equation:

$$\begin{array}{c} MN=\frac{APE\;in\;RN}{APE\;in\;MN}\times RN \end{array}$$

where APE in RN = the percent excess of ^15^N in the fraction analyzed, and APE in FPA microbial pellet was used as the source of APE in MN. Total MN production (mg/day) was calculated as the sum of microbial production in the eﬄuent, FPA, FPB of straw residues, and FPB of concentrate residues.

Totals presented in **Table 3** were calculated as [(concentrate + straw before incubation) – (concentrate + straw after incubation)]/(concentrate + straw before incubation).

**Table 3:** Effect of inoculum and ammoniation treatment (trt) of barley straw on DMD, OMD, TDMD, NDFD ADFD, N disappearance, and microbial N production.^1^

Item	Treatment^2^					*P*-value		
	Untreated		AFEX		SEM	Trt^3^	Inoculum	Int^3^
	Slow	Fast	Slow	Fast				
**DMD (g/kg DM)**								
Barley straw	461^c4^	464^c^	612^b^	636^a^	5.9	<0.001	<0.001	0.002
Concentrate	846	816	848	848	15.2	0.079	0.11	0.11
Total	624	618	720	733	8.2	<0.001	0.46	0.052
**OMD (g/kg DM)**								
Barley straw	466^c^	467^c^	615^b^	639^a^	4.7	<0.001	<0.001	<0.001
Concentrate	875	854	879	875	12.7	0.12	0.14	0.31
Total	586^b^	580^b^	694^a^	707^a^	7.7	<0.001	0.32	0.021
**TDMD (g/kg DM**								
Barley straw	500^c^	503^c^	633^b^	666^a^	5.6	<0.001	< 0.001	<0.001
Concentrate	860	828	858	859	14.0	0.081	0.12	0.092
Total	607^c^	601^c^	700^b^	725^a^	7.3	<0.001	0.015	<0.001
**NDFD (g/kg DM)**								
Barley straw	455^c^	451^c^	559^b^	593^a^	9.4	<0.001	<0.001	<0.001
Concentrate	785^a^	740^b^	769^ab^	785^a^	21.7	0.23	0.23	0.022
Total	507^c^	498^d^	599^b^	627^a^	10.8	<0.001	0.004	<0.001
**ADFD (g/kg DM)**								
Barley straw	427^c^	441^c^	534^b^	577^a^	12.3	<0.001	<0.001	0.008
Concentrate	689^a^	649^b^	674^ab^	685^a^	18.2	0.35	0.20	0.028
Total	460^c^	465^c^	551^b^	585^a^	12.4	<0.001	<0.001	<0.001
**N Disappearance (g/kg DM)**								
Barley straw	681	643	773	759	10.9	<0.001	0.029	0.27
Concentrate	931	915	925	940	16.8	0.33	0.94	0.13
Total	852	827	846	849	14.4	0.33	0.19	0.11
**Microbial N production (mg/d)**								
Eﬄuent	31.1	34.5	29.7	36.2	2.50	0.94	0.048	0.52
FPA^5^	5.6	5.6	5.7	5.1	0.20	0.39	0.12	0.25
FPB straw	19.9	18.7	18.2	18.8	0.56	0.19	0.61	0.14
FPB concentrate	1.3	1.4	1.2	1.4	0.19	0.62	0.22	0.94
Total	57.6	60.2	54.8	63.3	2.73	0.95	0.045	0.27

*^1^DM, dry matter; DMD, dry matter disappearance; OMD, organic matter disappearance; NDFD, neutral detergent fiber disappearance; ADFD, acid detergent fiber disappearance; TDMD, true dry matter disappearance.*

*^2^Fast refers to inoculum from animals with fast rate of NDFD; slow refers to animals with slow rate of NDFD.*

*^3^Trt refers to straw treatment: AFEX or untreated; Int refers to interaction between treatment and inoculum.*

*^4^‘a, b, c, and d’ Values within a row with the same letter do not differ (*P* > 0.05).*

*^5^FPA, feed particle associated; FPB, feed particle bound.*

Relative bacterial populations were calculated as (total copy number of species in a given fermenter on a given day/total bacterial copy number in the same fermenter on the same day) × 100.

All data were analyzed using the MIXED procedure of SAS (SAS Inc., Cary, NC, USA). Individual fermenter considered the experimental unit with day of sampling treated as a repeated measure. Straw, inoculum, straw × inoculum were considered fixed effects while apparatus was considered a random effect. For each parameter analyzed a covariance structure among compound symmetry, heterogeneous compound symmetry, autoregressive, heterogeneous autoregressive, Toeplitz, unstructured, and banded was chosen based on the lowest corrected Akaike information critical values. Significance was declared at *P* < 0.05 and a trend was considered at 0.05 ≤*P* ≤ 0.10. Differences among treatments were determined using Fisher’s protected (*P* < 0.05) LSD test using the PDIFF option in SAS for straw × inoculum interactions.

## Results

### Disappearance and Fermentation Characteristics

Ammonia Fiber Expansion treated straw had greater DMD, OMD, TDMD, NDFD, and ADFD (*P* ± 0.001) than untreated straw (**Table 3**). The straw × inoculum interactions (*P* < 0.05) for these variables indicate that Fast inoculum increased (*P* < 0.05) disappearance of AFEX straw, but had no effect on untreated straw. The NDFD and ADFD of concentrate was lowered with Fast inoculum with untreated straw (*P* < 0.05), but was not affected by the other treatments.

The N disappearance was greater (*P* < 0.001) for AFEX straw than for untreated straw (**Table 3**). N disappearance of untreated straw increased with Slow inoculum (*P* = 0.008), but inoculum source had no effect on N disappearance from AFEX. Microbial N production was greater for Fast inoculum in the eﬄuent and overall (*P* < 0.05).

Untreated straw produced more CH_4_ per gram of DMD then did AFEX straw (*P* = 0.046; **Table 4**). No other CH_4_ variable was affected by straw or inoculum source. AFEX straw decreased pH compared to untreated straw (*P* < 0.001). AFEX straw and Slow inoculum promoted greater NH_3_N production than untreated straw (*P* < 0.001) and Fast inoculum (*P* = 0.015), with no interaction between the two. AFEX straw resulted in more total VFA production than untreated straw (*P* < 0.001), and the straw × inoculum interaction indicated that more VFA was produced with AFEX straw incubated with Fast inoculum (*P* = 0.035) whereas Fast inoculum had no effect on VFA from untreated straw. Interactions were also observed for the proportions of acetate, butyrate, and caproate (*P* < 0.05). Fast inoculum decreased the proportion of acetate for untreated straw (*P* < 0.001) and caproate for AFEX straw (*P* < 0.001). Fast inoculum increased the molar proportion of butyrate for untreated straw, yet it increased it for AFEX straw (*P* < 0.001), although proportions were greater for untreated than AFEX straw. AFEX increased (*P* < 0.001) the molar proportion of propionate, but reduced (*P* < 0.001) that of valerate, isobutyrate, and isovalerate. AFEX also reduced the A:P ratio (*P* < 0.001), with the effect of inoculum dependent on straw type; Fast inoculum reduced (*P* = 0.029) A:P ratio for untreated, but not AFEX straw.

**Table 4:** Effect of inoculum and ammoniation treatment (trt) of barley straw on gas production and fermentation variables (pH, total VFA and individual VFA).

Item	Treatment^1^					*P*-value		
	Untreated		AFEX		SEM	Trt^2^	Inoculum	Int^2^
	Slow	Fast	Slow	Fast				
**Gas production**								
Total, L/d	1.54	1.61	1.59	1.60	0.135	0.87	0.76	0.79
CH_4_, %	6.20	6.37	6.14	6.11	0.534	0.66	0.88	0.79
CH_4_, mL/d	94.2	101	94.1	105	14.4	0.83	0.23	0.83
CH_4_, mg/d	61.2	66.0	61.2	67.6	9.30	0.87	0.26	0.86
CH_4_, mg/g incubated DM	6.66	7.19	6.65	7.32	0.973	0.91	0.26	0.88
CH_4_, mg/g digested DM	11.7	12.9	9.67	10.8	1.49	0.046	0.22	0.98
pH	6.70	6.69	6.64	6.61	0.019	<0.001	0.095	0.34
Ammonia, mg/d	110	98.3	137	131	5.29	<0.001	0.015	0.45
Total VFA, mmol/d	50.6^c3^	49.0^c^	57.4^b^	59.6^a^	1.88	<0.001	0.67	0.035
VFA, mol/100 mol								
Acetate (A)	66.1^a^	63.9^b^	63.7^b^	64.1^b^	0.618	0.001	0.006	<0.001
Propionate (P)	22.6	23.8	25.9	26.2	0.603	<0.001	0.021	0.21
Butyrate	7.22^b^	8.28^a^	6.74^c^	6.17^d^	0.098	<0.001	0.025	<0.001
Valerate	1.62	1.66	1.40	1.34	0.042	<0.001	0.60	0.10
Isobutyrate	0.916	0.933	0.849	0.848	0.012	<0.001	0.55	0.48
Isovalerate	1.51	1.44	1.33	1.30	0.035	<0.001	0.19	0.52
Caproate (×10^-2^)	4.52^c^	4.59^c^	5.19^a^	4.98^b^	0.011	<0.001	0.21	0.018
A:P ratio	2.93^a^	2.69^b^	2.47^c^	2.45^c^	0.090	<0.001	0.012	0.029

*^1^Fast refers to inoculum from animals with fast rate of NDFD; slow refers to animals with slow rate of NDFD.*

*^2^Trt refers to straw treatment: AFEX or untreated; Int refers to interaction between treatment and inoculum.*

*^3^‘a, b, c, and d’ Values within a row with the same letter do not differ (*P* > 0.05).*

### Microbial Populations

Ammonia Fiber Expansion had no effect on total protozoa counts, nor was there an effect (*P* > 0.10) on bacterial populations (**Table 5**). Copy numbers of *R. albus* were increased (*P* = 0.035) for Fast inoculum. Total bacterial 16S rRNA after adaptation tended to be greater (*P* = 0.10) for AFEX straw, with Fast inoculum increasing copies for AFEX but not untreated straw (interaction, *P* = 0.013).

**Table 5:** Effect of inoculum and ammoniation treatment (trt) of barley straw on rumen microbes.^1^

Item	Treatment^2^					*P*-value		
	Untreated		AFEX		SEM	Trt^3^	Inoculum	Int^3^
	Slow	Fast	Slow	Fast				
Total protozoa, × 10^3^ cells/mL	8.58	8.00	6.67	6.25	1.43	0.22	0.73	0.95
Total bacterial 16S rRNA copies after adaptation (×10^9^)^4^	107.9^ab5^	85.9^b^	96.8^b^	130.0^a^	14.5	0.10	0.56	0.013
*Fibrobacter succinogenes* (%)	2.41	1.29	2.17	1.25	0.718	0.85	0.18	0.89
*Ruminococcus albus* (%)	0.0286	0.0433	0.0258	0.0522	0.0087	0.74	0.035	0.51
*Ruminococcus flavefaciens* (%)	0.0678	0.0538	0.0585	0.0610	0.0095	0.91	0.56	0.41
*Selenomonas ruminantium* (%)	0.0422	0.0685	0.0599	0.108	0.041	0.37	0.25	0.73
*Prevotella bryantii* (%)	0.0186	0.0011	0.0156	0.0323	0.0165	0.40	0.98	0.31

*^1^Populations calculated as percent of total bacterial 16S rRNA.*

*^2^Fast refers to inoculum from animals with fast rate of passage; slow refers to animals with slow rate of passage.*

*^3^Trt refers to straw treatment: ammoniated or untreated; Int refers to interaction between treatment and inoculum.*

*^4^All bacteria quantified using FPA samples from each fermenter.*

*^5^Letters ‘a, b, c, and d’ denote significant difference, values with the same letter are not significantly different than each other.*

## Discussion

Ammoniation is known to increase DMD and N content of various straws including wheat (Horton, 1981; Herrera-Saldana et al., 1982; Givens et al., 1988; Kondo et al., 1992), oat (Horton, 1981; Givens et al., 1988), and barley (Horton, 1981; Hadjipanayiotou, 1982; Dryden and Kempton, 1983; Givens et al., 1988). AFEX is an advanced ammoniation technology and has been shown to increase digestibility of crop residues, compared to traditional ammoniation, by cleaving the hemicellulose-lignin ester linkages, or lignin carbohydrate complexes more efficiently (Chundawat et al., 2010). Ammoniation treatment disrupts the crystalline structure of cellulose I converting it to cellulose III (Mittal et al., 2011), which allows for much faster hydrolysation of β1–4 glycosidic bonds by microbial enzymes (Fan et al., 1980; Igarashi et al., 2007; Hall et al., 2010). Dale et al. (1997) found even low levels of enzymes digested AFEX to near theoretical yields. Thus, the 26% greater DMD and 21% greater NDFD of AFEX compared with untreated barley straw observed in the present study is consistent with the previous literature, and highlights the potential of AFEX technology to improve nutritive value of straw for feed. While AFEX may be impractical to implement on farms, Campbell et al. (2013) are working on developing this technology for regional depots, which would greatly increase access to this technology.

Observed differences between Fast and Slow inoculums may be attributed to differences in microbial populations within the inoculum, as the Rusitec system removes variation in physiological factors between ruminants such as saliva production, rumen fill, rumination time, rate of passage, and rate of absorption, that contribute to individual variability in fiber digestion observed *in vivo*. The increase in DMD, TDMD, and ADFD of AFEX straw when incubated with Fast inoculum in the Rusitec, with no effect on disappearance of untreated straw, indicates that heifers selected based on faster rate of NDF digestion of untreated barley straw were able to more thoroughly digest AFEX straw in 48 h compared to animals selected for slower rate of digestion. Rumen inoculum selected on the basis of a faster rate of NDF disappearance would likely contain greater populations and activity of microorganisms that degrade cell wall, which is consistent with the observation that *R. albus* was more abundant in Fast rumen inoculum than in Slow rumen inoculum. *R. albus* has long been known to be one of the most cellulolytic organisms in the rumen (Graham et al., 1985). The lack of effect of rumen fluid on the populations of the other four bacteria characterized may simply indicate that they were not responsible for the differences in digestibility observed. There are many fibrolytic bacteria such as *Butyrivibrio fibrisolvens, Clostridium longisporum, Clostridium lochheadii, Eubacterium cellulosolvens*, and *Prevotella spp.* (Stewart et al., 1997) that were not characterized in this study. This study did not look at interactions between bacteria, for example *Prevotella spp.* are known to be very effective at digesting hemicellulose in alfalfa, as well as contributing to increased digestion of cellulose when cultured with other cellulolytic bacteria (Dehority and Scott, 1967). There are also many, as of yet unculturable bacteria, that may also contribute to differences in ruminal degradation. Pooling the rumen fluid from two animals may have also eliminated some of the differences between individual Fast and Slow inoculum in relative population size of the selected bacteria due to potential antagonistic differences between bacteria from each donor animal. In addition, differences in methanogens, fungi, and protozoa species that may have contributed to differences in digestion observed were not characterized.

The increase in N disappearance of barley straw seen for AFEX was likely due to increased accessibility of cell wall contents due to enhanced NDFD (Graham and Åman, 1984). Ammoniated straw also contained more N than untreated barley straw because N from the ammoniation treatment is bound by the forage during treatment. While this excess is reduced by the ammonia recovery step in AFEX treatment (Chundawat et al., 2013), some of the N remains bound to the substrate and AFEX straw which accounts for the greater initial N content of AFEX compared with untreated straw (99 versus 43 g/kg DM). This agrees with the findings of Bals et al. (2010) who found increased N compared to untreated substrate for corn stover and switchgrass but reduced N compared to traditional ammoniation. The increase in N available for use in the rumen, along with the increase in degradability of AFEX straw, make it appealing as a potential feedstuff for cattle.

The reduction in CH_4_ when expressed relative to digested DM for AFEX compared with untreated straw was likely due to greater propionate and decreased butyrate molar proportions, and lower acetate to propionate ratio. Propionate acts as an alternative hydrogen sink in the rumen diverting hydrogen away from CH_4_ synthesis while the production of butyrate and acetate promote methanogenesis (Moss et al., 2000). The increase in VFA production caused by the AFEX treatment was likely responsible for the slight, but significantly lower pH measured in those fermenters compared with those fed untreated straw.

This study focuses on differences in rumen inoculum, but it is well known that other characteristics of individual animals, such as rumination time, saliva production, rumen fill, rate of passage, and rate of absorption also impact their ability to digest forage. As we continue to demystify the interactions between host animals and their microbiome, improving the ability of individual animals to digest forages will become more tenable.

## Conclusion

Ammonia Fiber Expansion was found to increase digestibility of barley straw DM by more than 30%. As AFEX technology becomes more widely available, AFEX treatment of straw has potential to increase straw usage as feed. Further to this, research is ongoing on microbiome contributions to variations in metabolic efficiency among animals (e.g., Hernandez-Sanabria et al., 2011; Khiaosa-ard and Zebeli, 2014). These differences may one day be exploited to improve individual efficiency. In working toward this, our study showed that rate of digestion due to rumen fluid source can be an important differentiating factor among ruminants, and contribute to significant differences in their ability to digest forage. This is likely due to differences in microbial populations, although this cannot be confirmed based on this study due to the limited number of bacterial species examined. In trying to improve the ability of ruminants to digest fiber it will be important to explore both physiological and microbiome characteristics of individual animals, and their interactions.

## Author Contributions

CG conducted all aspects of the study and drafted the manuscript; GR helped conduct the study, contributed to data analysis and revised the manuscript; and MO, TM and, KB provided advice on the protocol, data analysis, and final manuscript.

## Conflict of Interest Statement

CG received scholarships from Agriculture and Agri-Food Canada and the Canadian Dairy Commission and GR received a post-doctoral fellowship from the CAPES Foundation of the Ministry of Education of Brazil. Agriculture and Agri-Food Canada received partial funding from Elanco Animal Health to conduct the research. No patents or copyrights arose from the research. All the other authors declare that the research was conducted in the absence of any commercial or financial relationships that could be construed as a potential conflict of interest.
